# Rapid habituation of a touch-induced escape response in Zebrafish (*Danio rerio*) Larvae

**DOI:** 10.1371/journal.pone.0214374

**Published:** 2019-04-04

**Authors:** Adam C. Roberts, Julia Chornak, Joseph B. Alzagatiti, Duy T. Ly, Brent R. Bill, Janie Trinkeller, Kaycey C. Pearce, Ronny C. Choe, C. S. Campbell, Dustin Wong, Emily Deutsch, Sarah Hernandez, David L. Glanzman

**Affiliations:** 1 Department of Psychology, California State University at Fullerton, Fullerton, CA, United States of America; 2 Department of Neuroscience, University of California, Los Angeles, Los Angeles, CA, United States of America; 3 Department of Integrative Biology and Physiology, University of California, Los Angeles, Los Angeles, CA, United States of America; 4 Department of Biology, University of Texas at Tyler, Tyler, TX, United States of America; 5 Department of Neurobiology, David Geffen School of Medicine at UCLA, Los Angeles, CA, United States of America; 6 Integrative Center for Learning and Memory, Brain Research Institute, David Geffen School of Medicine at UCLA, Los Angeles, CA, United States of America; National Institutes of Health, UNITED STATES

## Abstract

Zebrafish larvae have several biological features that make them useful for cellular investigations of the mechanisms underlying learning and memory. Of particular interest in this regard is a rapid escape, or startle, reflex possessed by zebrafish larvae; this reflex, the C-start, is mediated by a relatively simple neuronal circuit and exhibits habituation, a non-associative form of learning. Here we demonstrate a rapid form of habituation of the C-start to touch that resembles the previously reported rapid habituation induced by auditory or vibrational stimuli. We also show that touch-induced habituation exhibits input specificity. This work sets the stage for *in vivo* optical investigations of the cellular sites of plasticity that mediate habituation of the C-start in the larval zebrafish.

## Introduction

Zebrafish larvae are emerging as an important model organism for gaining biological insights into behavior [1]. Interest in the larval zebrafish has been fueled, in part, by the availability of powerful reverse genetic techniques for disrupting gene function [2–7] and for modifying gene expression in this organism [8]. In addition, forward genetic screens are being developed to identify genes that play critical roles in behavior in larval zebrafish [9]. Besides these genetic advances, the amenability of zebrafish larvae, due to their translucency, to optogenetic manipulation and monitoring of neuronal activity *in vivo* [10–13], make these animals particularly attractive for addressing fundamental questions regarding the biology of learning and memory. Toward this end, it is important to develop learning protocols for the zebrafish larvae that are suitable for use with optical techniques. Accordingly, we have succeeded in demonstrating a form of habituation of a simple, neurobiologically tractable, behavior in the restrained zebrafish.

Brief auditory pulses elicit a short-latency startle response, the C-start, in zebrafish; this response results from selective activation of one of a pair of large hindbrain command neurons, the Mauthner cells (M-cells) [14]. By contrast, a touch can activate either the M-cell or M-cell analogs, depending on the fish’s developmental stage and the location of the skin stimulation [15]. Audiogenic startle responses are largely elicited through activation of the 8^th^ nerve, which forms highly specialized synapses, known as club synapses, on the distal lateral dendrites of the M-cell [16]. These club synapses are electrochemical; current transfer occurs through both gap junctions and glutamatergic synaptic transmission [17]. Stimulation of the larval zebrafish head through touch or electrical shock, on the other hand, induces activation of the trigeminal sensory neurons [18], which form exclusively chemical (glutamatergic) synapses onto the M-cell’s proximal lateral dendrite [16]. Although there have been several reports of habituation of the acoustic C-start (aC-start) [19–21], habituation of the touch-induced C-start (tC-start) has not been previously shown. Here we report the first demonstration of the (tC-start). As we show, this form of behavioral plasticity is at least partially pathway specific and may depend on glycinergic transmission.

## Materials and methods

### Animals and behavioral apparatus

Standard breeding protocols were used in accordance with UCLA Animal Research Care (ARC) requirements (fulfilled and approval given for research, IACUC number: 2016-099-03B). Zebrafish eggs were collected, placed into E3 solution (5 mM NaCl, 0.33 mM MgSO_4_, 0.33 mM CaCl_2_, 0.17 mM KCl, 10^−5^% methylene blue, pH 7.2), and incubated at 28.5°C. All experiments were conducted with the Tüpfel long fin (TL) wild-type strain of zebrafish obtained from the University of California, Los Angeles (UCLA) core facility. A Casio Exilim ExFH25 (Casio America, Dover, NJ) was used for all behavioral experiments; images were recorded at 240 frames/s. During the experiments, fish were placed on a light box (Gagne Inc., Johnson City NY) to allow sufficient contrast to observe the escape responses.

### Behavioral methods

#### Sensory stimulation by electrical or water pulses

**Fig 1:**
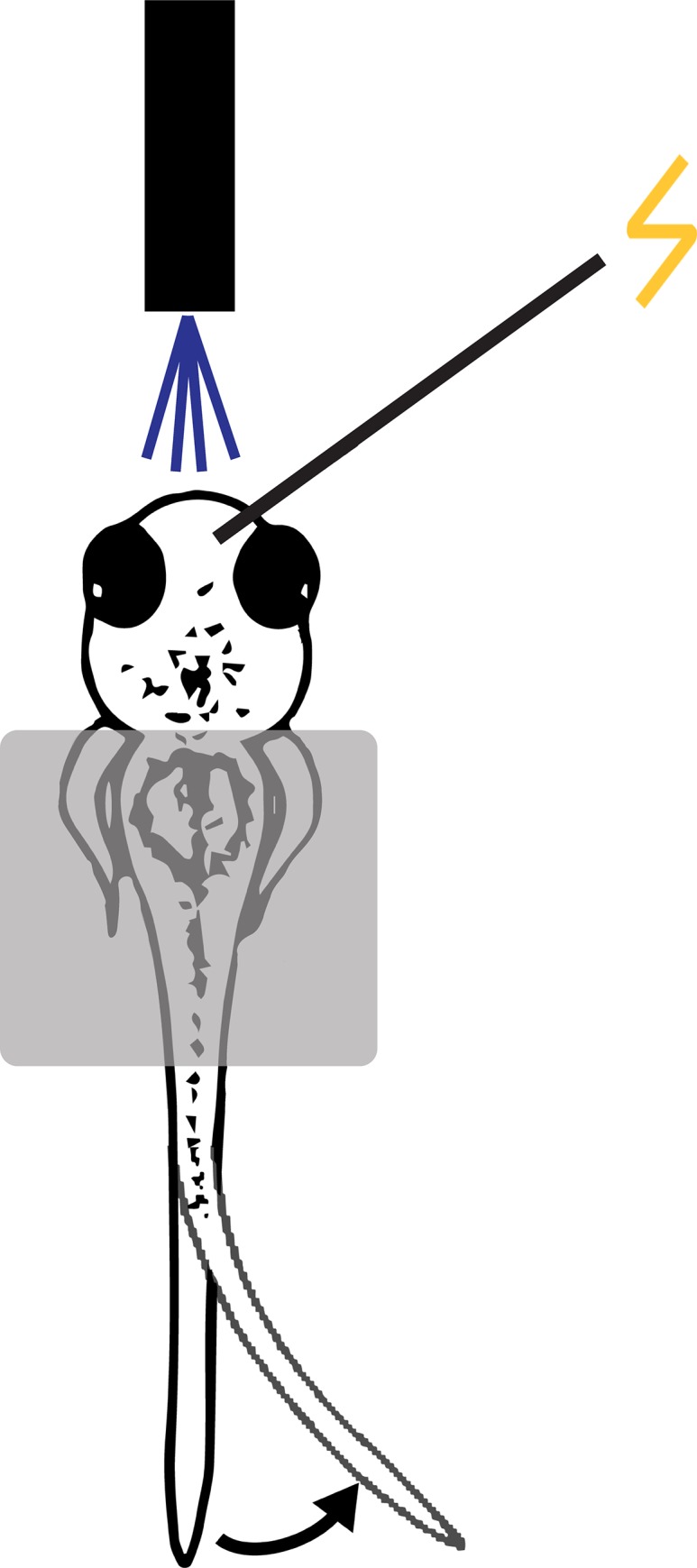
Experimental arrangement for habituating the touch-induced C-start in zebrafish larvae. Larval zebrafish (3–4 dpf) were placed in low melting point agarose. The agarose was removed from the tail to allow it to move freely. Agarose was also removed from part of the head to allow for electrical or mechanosensory stimulation of the head. Responses of the larvae to either electrical stimulation or water pulses were recorded using a high-speed video camera.

A semi-restrained preparation was used. Here, 3–4 days post fertilization (dpf) larval zebrafish were embedded in 3.5% low melting point agarose in a cell culture dish. After the agarose had solidified, E3 (or drug-containing E3) was added to the dish. The tail of the fish was freed from the agarose to permit it to move, and a portion of agarose above the head was removed to permit electrical or mechanosensory stimulation of the head (Fig 1). In experiments using electrical stimulation, larvae were acclimated for 45 min and startle thresholds—defined as the lowest current required to elicit a C-start—were determined prior to the experiment. The electrical stimuli (1-ms pulses at 500 Hz, 5-ms train duration) were delivered via one or more bipolar electrodes (two-conductor cluster electrode, 25 μM diameter; FHC Inc., Bowdoin, ME), which were placed on the skin of the larva’s head, anterior to the hindbrain. During experiments, shocks—20% above the threshold value (0.003–0.039 amps)—were delivered to the skin of the top of the head. For mechanosensory stimulation, a glass electrode was broken at the tip, and water from the bath was drawn into the electrode through capillary action. Pulses of water (2–5 ms, 3–12 PSI) were ejected toward the head using a picospritzer (General Valve Corporation, Fairfield, NJ). The threshold for mechanosensory stimulus was initially determined and defined as the lowest combination of duration and pressure of the water pulse that elicited an escape response. During the experiments, the head was stimulated with water pulses whose force was 20% above threshold.

#### Habituation training protocol

The same stimuli used to test the responsiveness of the larval zebrafish were used to induce habituation. After allowing 30 min for the fish to acclimate to the experimental situation, the threshold intensity for eliciting the C-start was determined (see Behavioral methods); 15 min later habituation training commenced. The training comprised 120 electrical/mechanosensory stimuli delivered at 1 Hz. The response of the fish—whether or not a C-start was elicited—to the first training stimulus was used as the pretest response; posttests were given 10 s–5 min after training, depending on the training protocol. Control fish received a single pretest and a single posttest stimulus delivered at the equivalent experimental times as the pretest and posttest for the trained fish.

#### Two electrode stimulation protocol

Two stimulating electrodes were placed on either the same side or opposite sides of the head of a larva. For same-side stimulation, electrodes were placed approximately 1.5 mm apart in order to stimulate different receptive fields of trigeminal sensory neurons. For both same-side and opposite-side placement, one of the two electrodes was used to deliver habituating stimuli, while the other electrode was used only for posttest stimulation (There was no pretest stimulation in these experiments). One of the electrodes (training or control) delivered a posttest stimulus 10 s after the end of training; a second posttest stimulus was delivered 5 s later via the other electrode. Which electrode was used for the 10-s posttest and which for the later posttest was counterbalanced between the training and control electrodes.

### Pharmacology

Larval zebrafish were treated through bath application with Strychnine hemisulfate salt (100 μM; Sigma, St. Louis, MO) or control vehicle solutions 18–24 h prior to testing unless otherwise indicated. Fish were maintained in the same solution throughout an experiment.

### Statistical analyses

Comparisons were performed using either *t*-tests or ANOVAs. Paired *t*-tests were used for within subject comparisons, whereas unpaired *t*-tests were used to compare different groups. Repeated measures ANOVAs were used to assess the significance of differences between groups that were tested at three different times. Significant interactions were further probed using a one-way ANOVA. Post-hoc analyses of significant ANOVAs were performed using Tukey HSD tests.

## Results

### Shocks or touches delivered to the head of semi-restrained zebrafish larvae induce short-lasting habituation of the C-start

**Fig 2:**
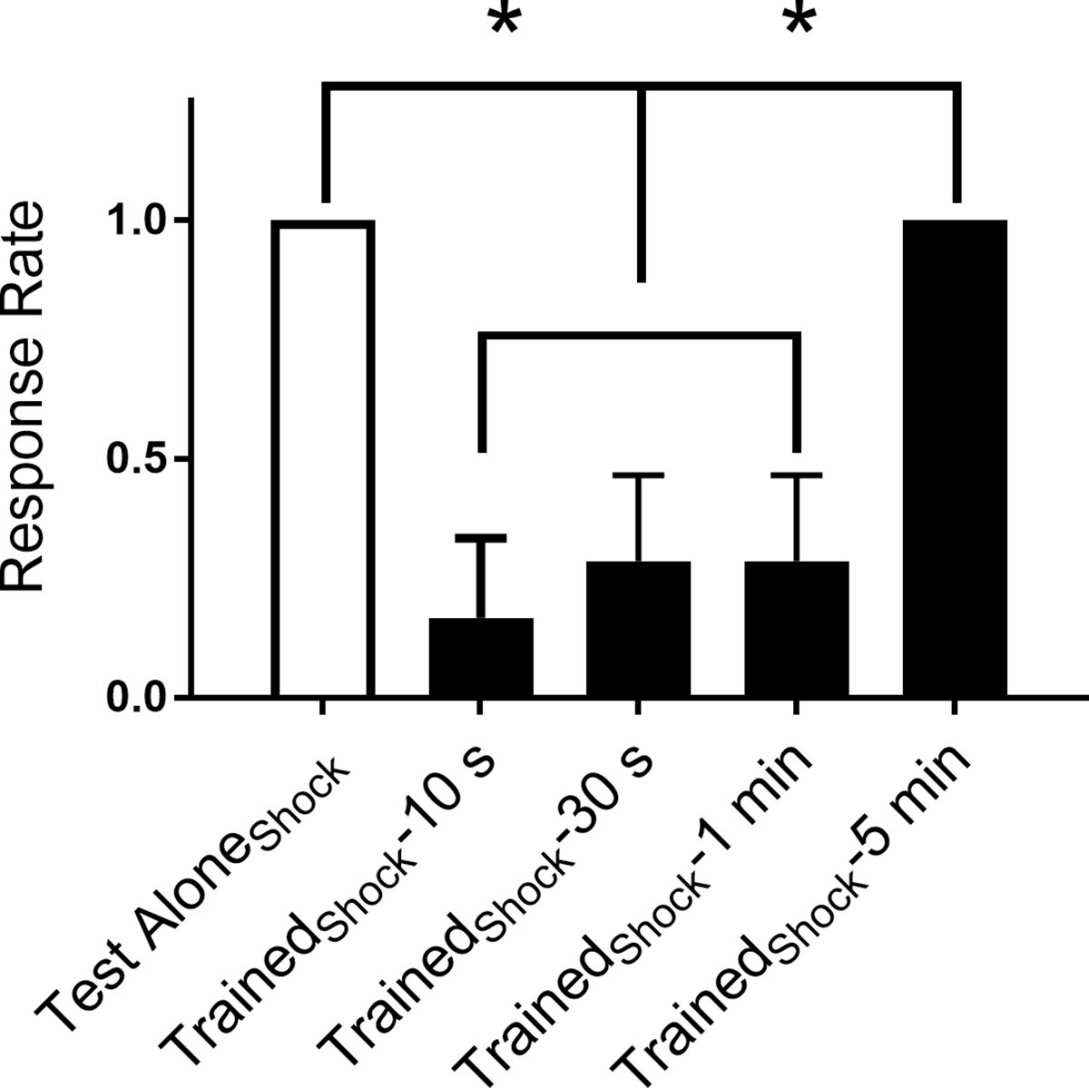
Rapid habituation of touch-induced C-start. Effect of electric shocks delivered at 1 Hz on the touch-induced C-start reflex. All groups, except one (Test Alone_Shock_, *n* = 7), were given posttests at various times after habituation training to determine the duration of the habituation with electric shocks. The Test Alone_Shock_ group received the test stimuli without habituation training. A between-groups ANOVA yielded a significant group effect (F_[__4__,__29__]_ = 9.22; *p* < 0.05). Tukey HSD post-hoc tests revealed that the Trained_Shock_-10 s group (*n* = 6), Trained_Shock_-30 s group (*n* = 7), and the Trained_Shock_-1 min (*n* = 7), were significantly less responsive than the Test Alone_Shock_ group (*n* = 7), and the Trained_Shock_-5 min (*n* = 7) group.

Two forms of behavioral plasticity of the C-start have been previously described in zebrafish larvae: auditory/vibrational (AV) stimulation-elicited habituation and dishabituation [1, 19–21]. AV stimulation selectively activates peripheral and central [16] neural circuitry distinct from that activated by stimulation of skin sensory receptors using head shock, water pulses, or touch. To determine whether stimuli that activate skin sensory receptors can elicit rapid habituation of the C-start reflex in larval zebrafish, we used a semi-restrained preparation. Habituation training consisted of 120 pulses of electrical shock delivered to the skin of the larva’s head. Posttests were performed 10 s, 30 s, 1 min, and 5 min after the end of habituation training. Only tail flips whose onset was < 25 ms after the stimulus were scored; such responses have been previously shown to represent C-starts [15, 22]. We observed rapid habituation of the C-start reflex (Fig 2). To exclude a potential contribution to habituation from repeated posttests, five groups of electric shock-trained/control fish were included. Each fish was given only a single posttest; however, different groups of trained/control fish received the posttest at different times after training (or at the equivalent experimental time in the case of the control animals). Electrical shocks elicited robust, but short-lasting, habituation. An ANOVA comparing fish habituated to electric shocks (Trained_Shock_-10 s, 0.17 ± 0.167; Trained_Shock_-30 s, 0.29 ± 0.184; Trained_Shock_-1 min, 0.29 ± 0.184; Trained_Shock_-5 min, 1.00 ± 0.000) and fish that only received the test stimulus (Test Alone_Shock_, 1.00 ± 0.00) revealed that the 10-s, 30-s, and 1 min posttest groups were each significantly different (*p* < 0.05 for each comparison) from the Trained_Shock_-5 min group and the Test Alone_Shock_ group. These data indicate that this form of habituation persists for 1 min, but not 5 min. Thus, the habituation induced was short-lasting. This form of habituation resembles the rapid form previously reported for AV stimulation [19–21].

**Fig 3:**
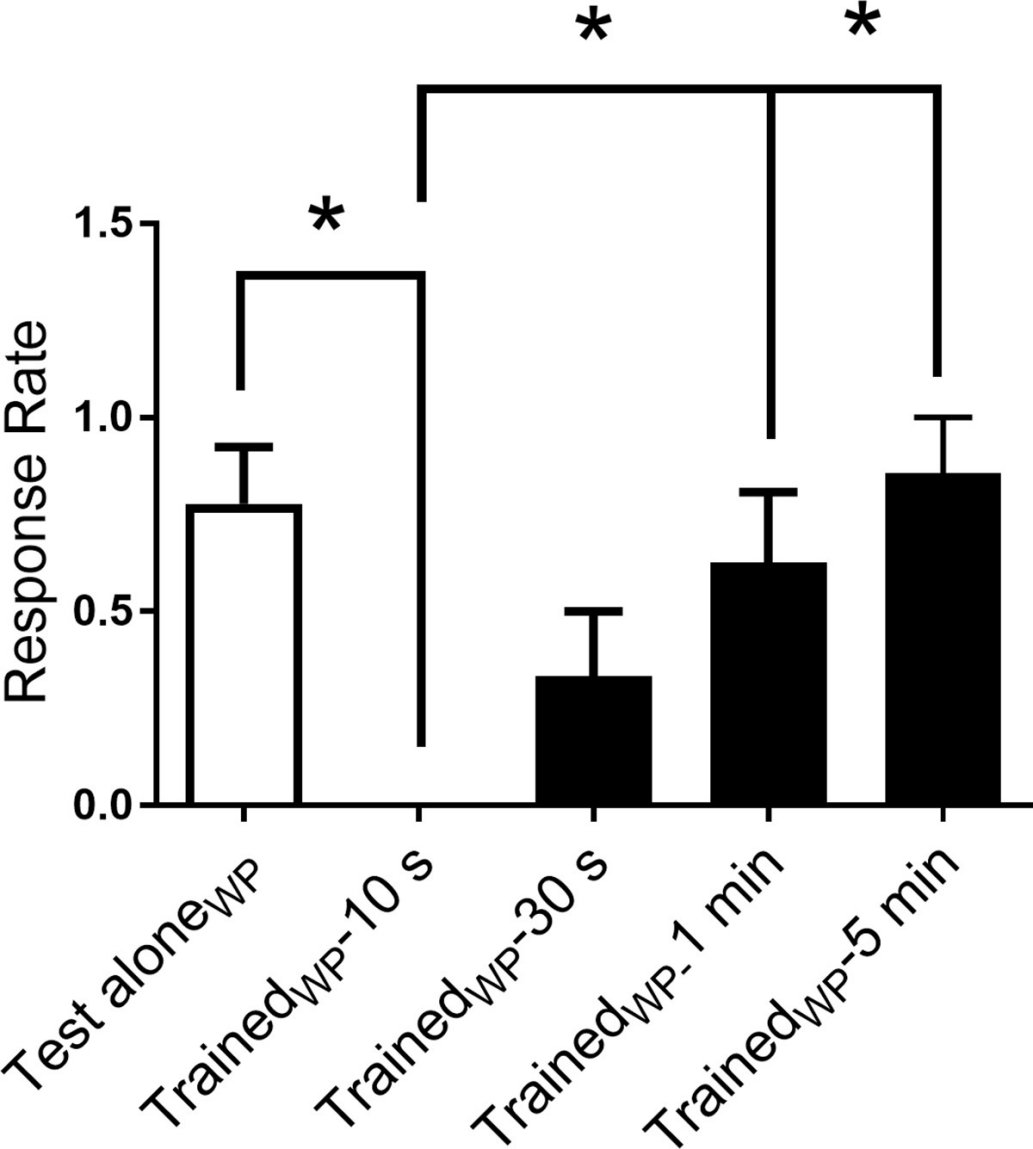
Stimulation of the head of larval zebrafish with water pulses induces rapid habituation. Effect of water pulses delivered at 1 Hz on the touch-induced C-start. All groups, except one (Test Alone_WP_, *n* = 9), were given posttests at various times after habituation training to determine the duration of the habituation with water pulses. The Test Alone_WP_ group received the test stimuli without habituation training. A between-groups ANOVA yielded a significant group effect (F_[__4__, 36]_ = 5.66; *p* < 0.05). Tukey HSD post-hoc tests revealed that the Trained_WP_-10 s group (*n* = 8) was significantly less responsive than the Test Alone_WP_ group (*n* = 9), the Trained_WP_-1 min (*n* = 8), and the Trained_WP_-5 min (*n* = 7) group. The Trained_WP_-10 s group was not statistically different from the Trained_WP_-30 s group (*n* = 9).

We also examined habituation of the startle response to water pulses [14, 22] (Fig 3). The tail-flip responses (onset less than 30 ms) of fish were measured at 10 s, 30 s, 1 min, and 5 min after the end of habituation training. To exclude a potential contribution to habituation from repeated posttests, five groups of water pulse (WP)-trained fish were included. A control group (Test Alone_WP_) received only the pretest and posttest. Groups of trained fish received the single posttest at various times after training (or at the equivalent experimental time in the case of the control animals). As observed in the experiments using electrical shocks, head-directed water pulses elicited robust, but short-lasting, habituation. An ANOVA comparing the response rate of fish given habituation training with water pulses (Trained_WP_-10 s, 0.000 ± 0.000; Trained_WP_-30 s, 0.333 ± 0.167; Trained_WP_-1 min, 0.625 ± 0.183; Trained_WP_-5 min, 0.857 ± 0.143) to fish that only received the test stimulus (Test Alone_WP_, 0.778 ± 0.147) revealed a significant difference at the 10-s posttest (*p* < 0.05) compared to all groups other than the Trained _WP_-30 s group. These data indicate that this form of habituation lasts for 10–30 s, but not longer.

### Shock-induced habituation of the C-start exhibits input specificity

**Fig 4:**
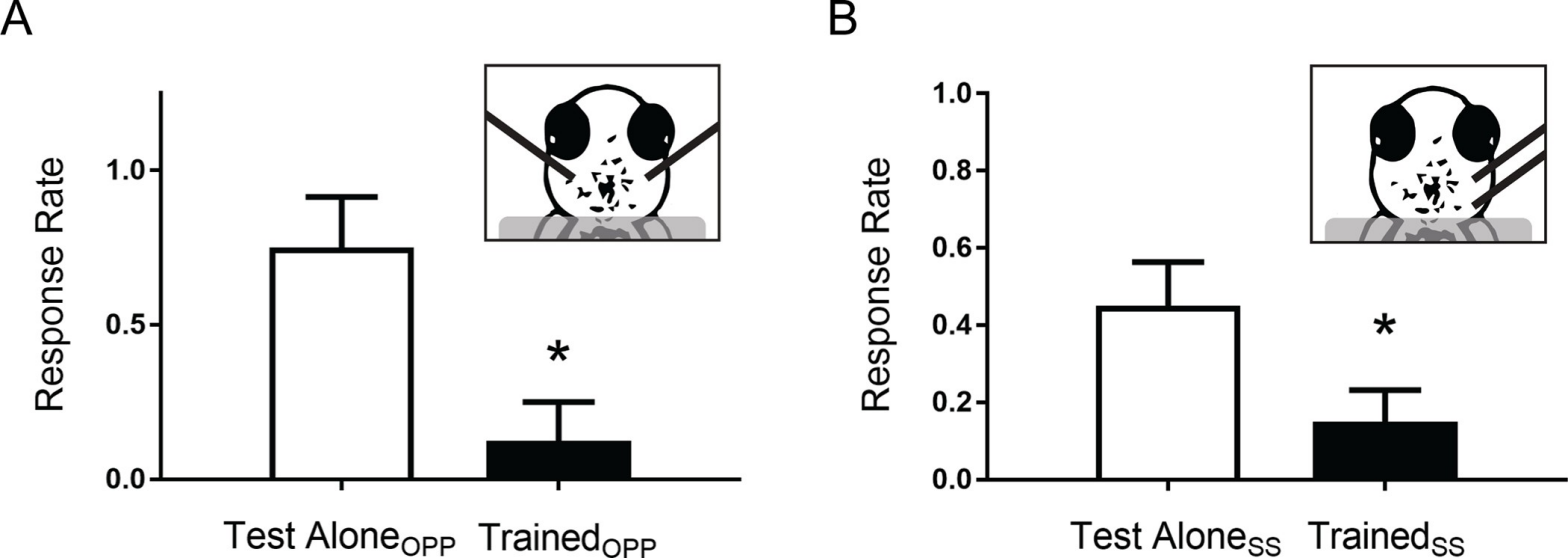
Rapid habituation of the touch-induced C-start reflex is circuit-specific. (a) Comparison of the response rate of restrained larvae given habituation training with electrical shocks to one side of the head (Trained_OPP_, *n* = 8) to their response rate to test stimulation alone of the opposite side (OPP) of the head (Test Alone_OPP_, *n* = 8). A paired *t*-test determined that the responsiveness of the trained side was significantly less than that of the untrained side (*t*_[__7__]_ = 3.42; *p* < 0.05). Long black bars in this and the following figure (part [b]) represent the tips of the extracellular electrodes. (b) Comparison of the response rates of larvae to habituating stimulation (Trained_SS_, *n* = 20) and to test stimulation alone delivered to the same side (SS) of the head (Test Alone_SS_, *n* = 20). The electrodes stimulated patches of skin whose sensory afferents synapsed onto the same M-cell. A paired *t*-test indicated that the response rates to the posttest stimuli differed significantly (*t*_[__19__]_ = 2.35; *p* < 0.05).

The habituation protocol we used elicits a significant, albeit transient, reduction in the startle response rate (Figs 2 and 3). This behavioral change may have resulted from input-specific habituation or, instead, from a stimulation-induced generalized decrease in the responsiveness of the fish. To determine which of these possibilities was the case, we performed additional experiments using two stimulating electrodes to selectively stimulate different touch-sensitive circuits. Trigeminal sensory neurons, the primary sensory neurons that innervate the skin of the larval zebrafish head, do not normally cross the midline of the head [18]. Accordingly, by placing bipolar stimulating electrodes on opposite sides of the head, we could selectively activate command neurons on either side of the brain [23]. (We confirmed prior to the onset of each experiment that the two electrodes evoked contralateral escape responses in a fish.) Stimulating electrodes were randomly assigned to deliver either habituation training followed by a posttest (Trained_OPP_) or only the posttest (Test Alone_OPP_). Posttests were delivered to the training and test alone sides of the heads in a counterbalanced manner to avoid order effects. Following habituation training, the Trained_OPP_ response (0.125 ± 0.125) was significantly (*p* < 0.05) reduced compared to the Test Alone_OPP_ response (0.750 ± 0.160) (Fig 4A). Thus, training using selective weak electrical stimulation of the skin on one side of the head induces habituation that results from unilateral alteration of neural circuits.

The habituation produced by unilateral skin shocks might have been due to pathway-specific homosynaptic depression or feedforward inhibition of the sensory-neuron-to-command-neuron circuit [24]; alternatively, the training stimulation might have triggered a non-specific increase in inhibition, or decrease in excitability, that unilaterally suppressed the firing of the command neurons. To determine which of these two general possible explanations for shock-induced habituation was more likely, we positioned two stimulating electrodes on the same side (SS) of a fish’s head, but at different locations (Fig 4B). One of the two electrodes was randomly selected to deliver the habituating stimulation (Trained_SS_), whereas the other delivered only the posttest stimulation (Test Alone_SS_). We observed that the posttest response to stimulation with the Trained_SS_ electrode (0.150 ± 0.080) was significantly less than that of the stimulation with the Test Alone_SS_ electrode (0.450 ± 0.110, *p* < 0.05). This result indicates that untrained neural circuit maintains significant responsiveness and that, therefore, the habituation induced by the electrical stimulation was partly, if not fully, input-specific.

### Role of glycinergic inhibition in shock-induced habituation

**Fig 5:**
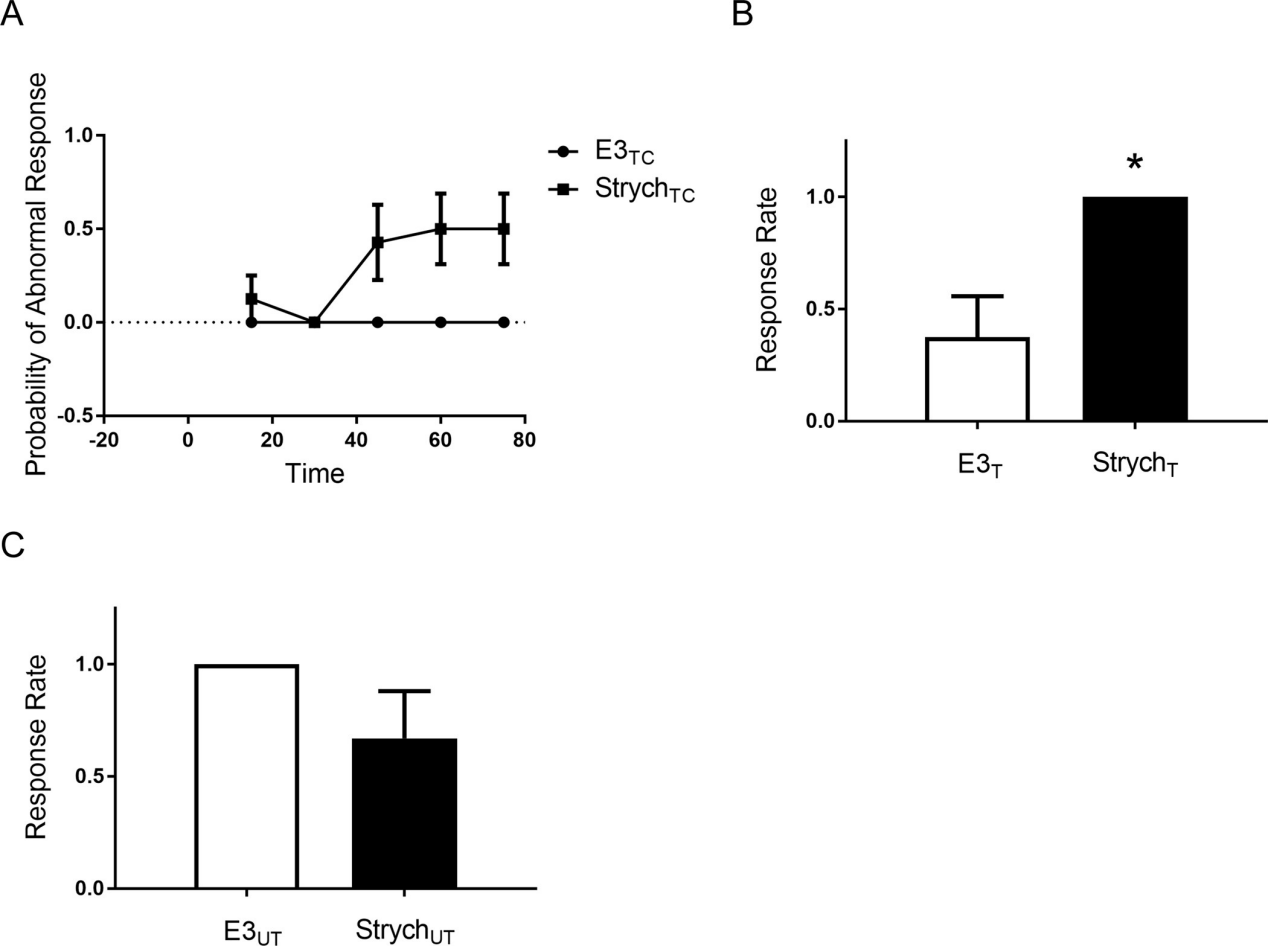
Strychnine blocks habituation to touch by disrupting the escape response. (a) Effect of strychnine (100 μM; Strych_TC,_ *n* = 7) or vehicle (E3_TC_, *n* = 6) on the nonhabituated escape response of larvae. A repeated measure between-groups ANOVA revealed a significant interaction between these groups (F_[__4__, 44]_ = 5.077; *p* < 0.05). Posthoc revealed that the differences in abnormal responses between the Strych_TC_ and E3_TC_ groups were significant for the 60-min (Strych_TC_, 0.50 ± 0.19; E3_TC,_ 0.00 ± 0.00), and 75-min posttests (Strych_TC_, 0.50 ± 0.19; E3_TC,_ 0.00 ± 0.00) (*p* < 0.05), but not for the 15-min (Strych_TC_, 0.13 ± 0.13; E3_TC,_ 0.00 ± 0.00), 30-min (Strych_TC_, 0.00 ± 0.00; E3_TC,_ 0.00 ± 0.00), or 45-min posttests (Strych_TC_, 0.43 ± 0.20; E3_TC,_ 0.00 ± 0.00) (*p* > 0.05). (b) Effect of strychnine on habituation of the escape response. To determine whether the altered startle response observed in strychnine-treated fish could still undergo habituation, we exposed groups to strychnine (Strych_T_, *n* = 6) or vehicle (E3_T_, *n* = 8) for 18–24 h prior to the onset of behavioral testing/training. One minute after habituation training, the Strych_T_ group was significantly (*t*_[__12__]_ = 2.93; *p* < 0.05) more responsive than the E3_T_ group. (c) Comparison of the effects of strychnine on fish that did not receive habituation training. The difference between the group treated with strychnine for 18–24 h (Strych_UT_, *n* = 8) and the group treated with vehicle (E3_UT_, *n* = 8) was not significant (*t*_[__14__]_ = 1.53; *p* = 0.15).

Marsden and Granato [25] reported that acoustic stimulation-induced, rapid habituation of the startle response in larval zebrafish depends on glycinergic inhibitory neurotransmission. Accordingly, we investigated whether rapid habituation of the C-start to electrical stimulation in the larval fish similarly involves inhibitory neurotransmission. The M-cell receives input from a large number of somatic glycinergic synapses [26, 27]; feed-forward enhancement [27, 28] of this inhibitory synaptic input might account for the reduction in escape responsiveness after habituation training. We used the glycine receptor antagonist strychnine to test for a potential role of glycinergic synaptic inputs in rapid habituation of the cutaneous receptor pathway. Strychnine has been previously shown to produce abnormal movements in response to tactile or acoustic stimuli in zebrafish embryos [25, 29], as well as to dramatically reduce habituation of acoustic startle [25]. We also observed that strychnine altered the escape response to a weak skin shock in larvae, changing it from a short-latency C-start to a short-latency spastic movement (Fig 5). (This abnormal response has also been referred to as accordion-like [29, 30]). Before testing the effects of strychnine on habituation, we determined how rapidly strychnine gains entry to the nervous system. For this purpose, we measured the quality of the shock-evoked startle response after bath application of strychnine at 15-min intervals. Responses were recorded and we determined if the short-latency responses were abnormal. We found that larvae exposed to strychnine (Strych_TC_ group) exhibited a significantly altered escape response within 60 min compared to a vehicle-treated control group (E3_TC_) (Fig 5A). Nonetheless, the larvae could still perform normal C-starts after a 1.5-h exposure to strychnine, which indicated that even after 1.5 h the drug’s penetrance into the CNS was incomplete. To ensure complete saturation of the central glycine receptors, larvae were therefore incubated for 18–24 h in strychnine prior to testing habituation of the escape response. Following this prolonged exposure to strychnine, the electrical stimuli did not evoke any normal C-starts in either the habituation-trained (Strych_T_) or untrained (Strych_UT_) larvae (Fig 5B and 5C). Eighteen-to-twenty-four h of strychnine exposure also significantly reduced habituation of the startle response in zebrafish larvae (probability of response = 1.00 ± 0.00 in the Strych_T_ group vs. 0.38 ± 0.18 in the E3_T_ group; *p* < 0.05) (Fig 5B). The difference in responsiveness in the group given only the test stimuli in the vehicle (E3_UT_ group, response rate = 1.00 ± 0.00) and that in the group given only the test stimuli after the strychnine treatment (Strych_UT_ group, response rate = 0.75 ± 0.16) was not significant (*p* = 0.15) (Fig 5C). These results support the idea that alterations in the level of glycinergic inhibition may mediate rapid habituation of the startle response [25]; nonetheless, the obvious abnormality of the tail-flip response after the prolonged exposure to strychnine argues for caution in interpreting the possible role of glycinergic inhibition in habituation in zebrafish larvae.

## Discussion

We have found that application of shocks or water pulses to the head of zebrafish larvae elicits a startle response from them whose short latency is consistent with mediation by an M-cell or M-cell analog [15, 19, 20, 22]. Furthermore, habituation of this reflex in 3–4 dpf larvae appears to have the same temporal dynamic previously observed in AV-elicited rapid habituation of the C-start reflex in 5 dpf larvae [19, 20]. Although we cannot rule out that our water pulse protocol inadvertently stimulated neuromasts or the otic vesicle, it should be noted that these sensory structures were fully or partially covered in agarose. Furthermore, the latencies of startle observed were delayed (16.7–29.2 ms) compared to those typically elicited by electrical shocks given in the bath or AV stimulation [31]. In addition, we confirmed a previous report [25] that treatment with strychnine disrupts habituation of the C-start. This finding supports the idea that glycinergic feedforward inhibition [27], as previously shown to be triggered by auditory stimulation [25], plays a role in habituation; nevertheless, this interpretation is complicated by the significant abnormality of the touch-induced responses produced by strychnine in the larvae. Therefore, additional experiments will be required to disambiguate the potential role of glycinergic neurotransmission in habituation in zebrafish larvae.

We also determined that habituation to weak head shocks is at least partly input-specific. Specifically, we showed that the habituation of the C-start to training on one side of the larva’s head did not generalize to the opposite side, as indicated by the lack of significant habituation to the opposite-side test stimulus (Fig 4A). On the other hand, same-side training did produce some generalization of habituation [32, 33]. The generalization of habituation could be ascribed to stimulation of partially overlapping sensory fields by the same-side electrodes. Alternatively, the generalization might have resulted from recruitment by the training stimulation of plasticity-inducing postsynaptic pathways within the Mauthner cell that partially reduced the strength of synapses made by sensory neurons activated by the test stimulus. Of course, other cellular mechanisms, for example, a short-term increase in tonic inhibition of the Mauthner cell, induced by the training, could also have contributed to the generalization of habituation.

In summary, the present study indicates that touch-induced habituation of the C-start reflex in zebrafish larvae exhibits input-specificity and generalization, two hallmarks of habituation [32, 33]. Moreover, the touch-induced habituation demonstrated here shares many of the properties of rapid habituation induced by AV stimulation [19, 20]. Finally, glycinergic inhibition may participate in this form of habituation. The experimental methods and training protocols developed here are suitable for optical investigations of habituation in the restrained larval zebrafish.

## Supporting information

S1 Fig Rapid habituation of the touch-induced C-start reflex is sensory modality-specific. (a) Comparison of the mean response rate of restrained larvae (*n* = 7) after habitation training with AV stimulation (Trained AV) to their mean response rate to the posttest electrical stimulus (Untrained StimElectrode). The response rate to the AV stimulus was significantly less than that to the electrical stimulus (paired *t-*test, *t*_[__6__]_ = 2.83; *p* < 0.05). (b) Comparison of the responsiveness of restrained larvae (*n* = 8) following habitation training with electrical shocks to the skin (Trained StimElectrode) to their responsiveness to the posttest AV stimulus (Untrained AV). The responsiveness of the larvae following the two types of experimental manipulation differed significantly (paired *t*-test, t _[__7__]_ = 3.42; *p* < 0.05).
(TIF)

S1 File Supporting methods and results. (DOCX)
