# Supplementary figures and images for: Rapid habituation of a touch-induced escape response in Zebrafish (Danio rerio) Larvae
Source: PLoS One. 2019 Apr 4;14(4):e0214374. doi: 10.1371/journal.pone.0214374 (PMC6449028; doi:10.1371/journal.pone.0214374)

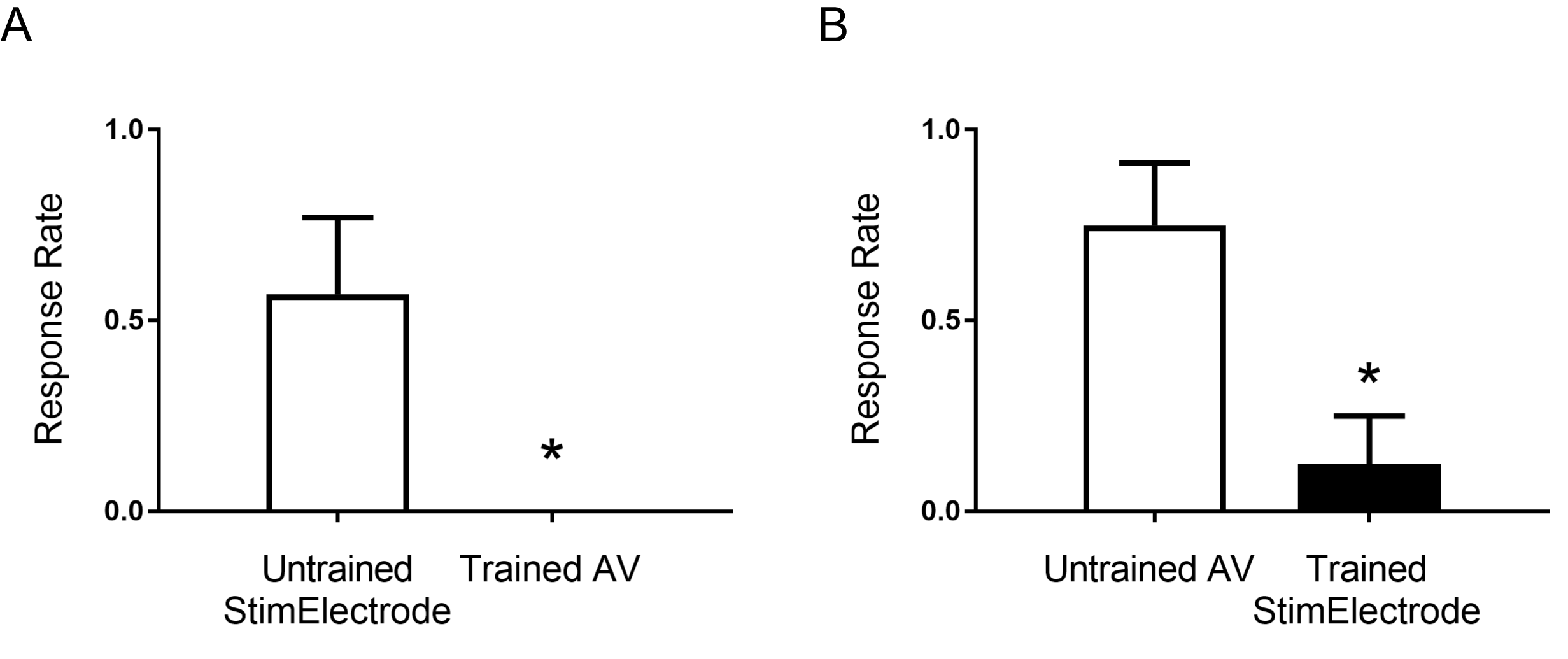

Supplement: S1 Fig — (a) Comparison of the mean response rate of restrained larvae (n = 7) after habitation training with AV stimulation (Trained AV) to their mean response rate to the posttest electrical stimulus (Untrained StimElectrode). The response rate to the AV stimulus was significantly less than that to the electrical stimulus (paired t-test, t [6] = 2.83; p < 0.05). (b) Comparison of the responsiveness of restrained larvae (n = 8) following habitation training with electrical shocks to the skin (Trained StimElectrode) to their responsiveness to the posttest AV stimulus (Untrained AV). The responsiveness of the larvae following the two types of experimental manipulation differed significantly (paired t-test, t [7] = 3.42; p < 0.05). (TIF) [file pone.0214374.s001.tif]
