# Supplementary material for: Rapid habituation of a touch-induced escape response in Zebrafish (Danio rerio) Larvae
Source: PLoS One. 2019 Apr 4;14(4):e0214374. doi: 10.1371/journal.pone.0214374 (PMC6449028; doi:10.1371/journal.pone.0214374)
Supplement: S1 File — (DOCX) [file pone.0214374.s002.docx]

**Supplementary methods**

Tüpfel long fin (TL) wild-type strain of zebrafish (5-6 dpf) were used for these supplementary experiments. A Casio Exilim ExFH25 (Casio America, Dover, NJ) was used to capture escape responses and images were recorded at 240 frames/s. After embedding the fish in agarose, the tail of the fish, a portion of the head, and one otic vesicle (that closer to a speaker placed on the light box) were freed to permit electrical or auditory/vibrational (AV) stimulation. A speaker (Visaton SC 5.9 with a Behringer A500 amplifier), which was placed ~ 5.5 cm from the center of the chamber containing the larval zebrafish, was used to elicit an AV startle response. The fish were initially placed on a light box and allowed to acclimate for 30 min, after which the threshold for eliciting startle via electrical stimulation of the skin was determined (see Materials and methods in the main text). Fifteen min after establishing the threshold, habituation training commenced. Habituation training (120 stimuli, 1 Hz) was given using either AV stimulation (82 dB; 2 ms pulse duration, 500 Hz) or electrical stimulation (20% above threshold, 0.005-0.012 amps; 500 Hz, 1 ms pulse duration, 5 ms train duration). Test pulses were given either 10 s or 15 s (counterbalanced between training and control stimuli) after the habituation training.

**Supplementary results**

Having demonstrated input specificity for habituation due to electrical stimulation of two different regions of skin, we tested whether habituation of two sensory pathways activated by qualitatively different types of sensory stimulation also exhibited input specificity. For this purpose, a bipolar stimulating electrode was placed on a patch of skin on the larva’s head; habituation could be elicited using two different types of sensory stimulation, electrical stimulation of the skin or AV stimulation. Stimulating electrodes were placed on the side of the head that was freed of agarose and closer to the speaker. When AV stimulation was used for habituation training, we observed a strong reduction in response rate (response rate = 0.00 ± 0.00) at the 10 s and 15 s posttest (S1a Fig.). By contrast, the responsiveness of larvae to posttest stimulation of the sensory pathway that did not receive habituation training (via the electrode) was significantly greater (response rate = 0.57 ± 0.20, *p* < 0.05). This result indicates that the habituation induced in the sensory pathway activated by the AV stimuli did not spread significantly to the sensory pathway activated by the electrical stimuli. Thus, habituation of the escape reflex in zebrafish larvae can be elicited in a sensory modality-specific manner.

Although highly unlikely given the size of the stimulating electrode and the demonstration of pathway specificity of habituation in experiments that used two stimulating electrodes (Fig. 4, main text), the strong posttest responsiveness to the electrical stimulus in the above experiment could arguably have been due to direct activation of either the M-cell or M-cell analogs. To rule out this explanation, we performed an additional experiment in which habituation training (120 stimuli, 1 Hz; intensity = 20% above threshold) was carried out via an electrode placed on the skin of the head. We observed a strong reduction in responsiveness (response rate = 0.13 ± 0.13) at the 10 s and 15s posttest to the electrical stimulus; moreover, the response rate to the posttest AV stimulus (0.75 ± 0.16) was significantly greater (*p* < 0.05) (S1b Fig.). An additional argument against the idea that the electrical stimulation used in our experiments directly elicited M-cell or M-cell analogs is that the latencies of the C-starts to the electrical stimuli (13.89 ± 1.39 ms) were relatively long. By comparison, Tabor et al. [1] found that C-starts elicited by direct electrical stimulation of the M-cell in zebrafish larvae (via bath stimulation using electrodes placed on either side of the head) had a much shorter latency (~ 2 ms). We confirmed that electrical stimulation of the bath elicits very short-latency C-starts, latencies that were significantly (*p* < 0.05) shorter than those elicited by the electrical stimulation of the skin (data not shown). Taken together, these results indicate that the skin shocks used in our habituation experiments did not elicit C-starts via direct M-cell/M-cell homolog activation.

**S1 Fig. Rapid habituation of the touch-induced C-start reflex is sensory modality-specific.** (a) Comparison of the mean response rate of restrained larvae (*n* = 7) after habitation training with AV stimulation (Trained AV) to their mean response rate to the posttest electrical stimulus (Untrained StimElectrode). The response rate to the AV stimulus was significantly less than that to the electrical stimulus (paired *t-*test, *t* _[6]_ = 2.83; *p* < 0.05). (b) Comparison of the responsiveness of restrained larvae (*n* = 8) following habitation training with electrical shocks to the skin (Trained StimElectrode) to their responsiveness to the posttest AV stimulus (Untrained AV). The responsiveness of the larvae following the two types of experimental manipulation differed significantly (paired *t*-test, t _[7]_ = 3.42; *p* < 0.05).

**Supplementary reference**

1. Tabor KM, Bergeron SA, Horstick EJ, Jordan DC, Aho V, Porkka-Heiskanen T, et al. Direct activation of the Mauthner cell by electric field pulses drives ultrarapid escape responses. J Neurophysiol. 2014;112(4):834-44. Epub 2014/05/23. doi: 10.1152/jn.00228.2014. PubMed PMID: 24848468; PubMed Central PMCID: PMCPMC4122749.
